# Validity of the self-reported five-part questionnaire as an assessment of generalized joint hypermobility in early pregnancy

**DOI:** 10.1186/s12891-020-03524-7

**Published:** 2020-08-03

**Authors:** Angela Schlager, Kerstin Ahlqvist, Ronnie Pingel, Lena Nilsson-Wikmar, Christina B. Olsson, Per Kristiansson

**Affiliations:** 1 Department of Public Health and Caring Sciences, Uppsala University, Husargatan 3, Box 564, 752 37 Uppsala, Sweden; 2 Department of Statistics, Uppsala University, Uppsala, Sweden; 3 Department of Neurobiology, Care Sciences and Society, Division of Physiotherapy, Karolinska Institutet, Huddinge, Sweden; 4 Academic Primary Healthcare Centre, Region Stockholm, Stockholm, Sweden

**Keywords:** Generalised joint hypermobility, Validity, Diagnostic accuracy, Beighton score, Five-part questionnaire

## Abstract

**Background:**

The assessment of generalized joint hypermobility is difficult due to differences in classification methods and in the performance of joint mobility assessment. The primary aim was to evaluate the validity of the self-reported five-part questionnaire, 5PQ, for identifying generalized joint hypermobility using the Beighton score as reference test. The secondary aim was to describe how joint angles measured in degrees included in the Beighton score varied in different cut-off levels in the self-reported 5PQ and the Beighton score.

**Methods:**

A cross-sectional validity study with a total of 301 women in early pregnancy, mean age of 31 years, were included in the study. The participants answered the self-reported 5PQ before the joint angles were measured. To standardize the joint mobility measurement, a structural protocol was used. The sensitivity, specificity, receiver operating characteristic curve, area under curve, positive- and negative predictive value, positive likelihood ratio and Spearman’s rank correlation between the self-reported 5PQ ≥ 2 and the Beighton score ≥ 5 were used as main outcome measures in the validity analyses. Joint angles, measured in degrees, were calculated with means in relation to different cut-off levels.

**Results:**

There was moderate correlation between the self-reported 5PQ and the Beighton score. The highest combined sensitivity, 84.1%, as well as specificity, 61.9%, was on 5PQ cut-off level ≥ 2, with a 38% false-positive rate, a moderate area under curve, a low positive predictive value and likelihood ratio, and a high negative predictive value. The odds of a self-reported 5PQ, cut-off level ≥ 2, among women with generalized joint hypermobility, Beighton ≥5, was low indicating a low post-test probability. The mean for all joint angles measured in degrees increased with increased cut-off levels, both in the Beighton score and in the self-reported 5PQ. However, there was a significant variation for each cut-off level.

**Conclusions:**

There is uncertainty in identifying generalized joint hypermobility in young women using the self-reported 5PQ with a cut-off level of ≥2 when the Beighton score ≥ 5 is used as the reference test. The strength of the self-reported 5PQ is to rule-out women without generalized joint hypermobility.

## Background

Generalized joint hypermobility (GJH) is a collagen phenotype that impacts the entire body and is the main criterion for heritable connective tissue disorders [1–3]. The definition is the ability of a group of joints, usually five or more, to move beyond their normal range of motion (ROM). GJH is part of the term joint hypermobility (JH). JH can also be divided into localized Joint Hypermobility (LJH) when JH exists at a single site, peripheral joint hypermobility (PJH), when JH exist in hands or feet or as historical joint hypermobility (HJH), the prior existence of JH in older adults who on clinical assessment have lost their JH [4, 5].

GJH can be asymptomatic but when symptomatic the clinical consequences are joint instability resulting in increased frequency of joint dislocation, subluxations, soft tissue overload, or injuries, defective neuromuscular control with reduced proprioception and balance, muscle weakness, prolonged and widespread musculoskeletal pain, pregnancy-related pelvic girdle pain, skin disorders, impaired effects of local anaesthesia, stomach disorders, and psychiatric disorders [2–4, 6–10]. Therefore, assessment of GJH is urged in clinical practice [1]. Although there is currently insufficient evidence, the clinical consequences might be relieved with a combination of physical therapy and pain management [11–13].

The prevalence of GJH in the general population is uncertain and seems to vary greatly. GJH is more common among women, in certain ethnic groups and decreases with increasing age body mass and waist circumference. The prevalence also depends on which assessment instruments and cut-off levels are used [14–18]. In Sweden, the prevalence of GJH among adults, mixed gender, assessed with a modification of the Beighton score with a cut-off value of three out of five, has been estimated to about 10% [19].

To clinically assess GJH, several hypermobility instruments have been developed using different sets of joints and different cut-off levels [2, 16].

There is as yet no consensus on a gold standard for assessing GJH. The Beighton score (BeS) is the most recognised instrument [4, 20] and is used in assessing GJH in hereditary connective tissue disorders, with a cut-off level of 5/9 for adults [2]. The BeS consists of a dichotomous assessment of five joint movements. The BeS is reliable and valid, but with conflicting results in studies due to differing methods and populations [16, 21–26]. A noted limitation of the BeS is the lack of assessment of common clinical sites of hypermobility, such as the cervical spine, shoulders, hips, and ankles [4]. However, for patients with a lower Beighton score, the assessment of other joints not included in the Beighton score is recommended [2], although the importance of these joints in GJH assessment needs further investigation. The Hospital del Mar criteria is a less commonly used instrument that also includes ball-and-socket joints, which might be beneficial in assessment of GJH [21].

To identify hypermobility, Hakim and Grahame constructed a self-reported instrument in the form of a five-part questionnaire (5PQ) that includes five aspects of past or present hypermobility [27]. The 5PQ correctly identified 84% of individuals with hypermobility with a cut-off level of two positive answers to any of the five questions with a sensitivity and specificity of 83 and 89%, respectively, using the revised Brighton criteria 1998 for Benign joint hypermobility syndrome, BJHS [28], as the reference test [27]. Previous studies showed that the self-reported 5PQ, cut-off level 2/5, is a valid and a reliable instrument to identify GJH, compared with the BeS with a cut-off level of 4/9 or 5/9, however, with populations that differed with respect to genders and ages [29, 30].

To identify, diagnose, and establish a rehabilitation plan for patients, valid and reliable diagnostic clinical assessment methods and criteria for classifying GJH are essential [31]. However, a recent systematic review stated that validity was insufficiently studied for the joint hypermobility instruments currently used [16]. Since the self-reported 5PQ and the BeS are the instruments predominantly used to identify GJH in clinical settings and in research, it is important to know if they identify GJH in a similar manner. A clinical examination of joints is time-consuming and the self-reported 5PQ offers a more practical way to identify GJH. It is also important to evaluate which cut-off level to use for each instrument.

This study is part of a longitudinal study of women during and after pregnancy, with the overall aim of investigating GJH and its relation to pregnancy-related pain. Therefore, it would be of value to investigate if the self-reported 5PQ for identifying GJH, could easily identify pregnant women with hypermobility.

The primary aim of this study was to evaluate the validity of the self-reported five-part questionnaire for identifying GJH, using the Beighton score as a reference test.

The secondary aim was to describe how the joint angles measured in degrees included in the Beighton score varied in different cut-off levels of the self-reported five-part questionnaire and the Beighton score.

## Methods

### Study design

This was a cross-sectional validity study.

The study is part of a longitudinal study of women during and after pregnancy, with the overall aim of investigating GJH and its relation to pregnancy-related pain.

### Setting

Pregnant women in Sweden have the right to regularly visit a maternal health care centre, free of charge. The centres are operated by the county councils, or are subcontracted by the councils. The centres are staffed by general practitioners, midwives, and administrative personnel.

### Study population

The majority of the 8029 women attending three maternal health care centres, two in Uppsala (5312) and one in Borlänge (2717), between February 2014 and December 2018, were invited to participate. Information about the study was given by their midwife or via a letter sent home. The women were consecutively recruited. A total of 339 pregnant women were included in the study (101 from Borlänge and 238 from Uppsala). All included women gave written informed consent to participate in the study. The inclusion criterion was duration of gestation less than 15 completed weeks and exclusion criteria were history of joint inflammation, spasticity, joint replacement, musculoskeletal injury in joints relevant for the assessment during the preceding 3 months, and not being fluent in the Swedish language. The duration of gestation was calculated after a midwife estimated date of birth.

### Data collection

Data collection was performed before gestational week 15. The women completed a web-based questionnaire covering age, completed weeks of gestation, BMI, marital status, education, country of birth, previous pregnancies, previous children and tobacco use. The women also completed the 5PQ. The self-reported 5PQ encompasses five aspects regarding past or present information on joint hypermobility. The questions are “Can you now (or could you ever) place your hands flat on the floor without bending your knees?”, “Can you now (or could you ever) bend your thumb to touch your forearm?”, “As a child, did you amuse your friends by contorting your body into strange shapes or could you do the splits?”, “As a child or teenager, did you dislocate your shoulder or kneecap on more than one occasion?” and “Do you consider yourself double-jointed?”. Each positive response adds 1 point, with a total score ranging from 0 to 5 [27]. In this study, all cut-off levels, 1–5, on the self-reported 5PQ were included in the evaluation.

### Clinical assessment

The BeS, used as the reference test [20], includes five assessments of joint movements: passive dorsiflexion of the fifth metacarpophalangeal joint ≥90 degrees, passive apposition of the thumb to the flexor aspects of the forearm, passive hyperextensions of the elbow and the knee ≥10 degrees, and forward flexion of the trunk with knees straight, with the palms of the hands rested on the floor*.* The first four are assessed bilaterally. A positive result in any assessment adds 1 point, with a total score ranging from 0 to 9. In this study, a cut-off level of ≥5 was used for identifying GJH according to the 2017 criteria to assess GJH in hereditary connective tissue disorders, with a cut-off level of 5/9 for adults [2]. All joint mobility measurements were performed in accordance with a structured protocol used in a previous study [32].

Three experienced physiotherapists and one experienced general practitioner working in primary care (termed raters from now on) performed all joint mobility measurements. To standardise performance and ensure similar assessments, the four raters trained un-blinded on three occasions, for a total of 24 h, with 21 people. The protocol, illustrated with photos, described start position, position of goniometer, anatomic landmarks, stabilisation of adjacent structures, and how to perform the test using active or passive movement. Two different goniometers (Medema Brodin, Kista, Sweden, 31 cm and 21 cm with a 180° protractor and movable arms) were used. The shorter goniometer was used for measurement of the fifth finger. Each joint angle was registered to the nearest 1 degree.

All measurements for each participant were performed on the same occasion. The participants filled out the 5PQ and provided their sociodemographic data, without the presence of the rater, before measurement of joint range of motion, ROM, was performed. The raters were blinded to the answers in the self-reported 5PQ during clinical examination.

The rater started each joint mobility assessment by providing the participant with verbal and visual instructions about the test procedure. No warm-up session preceded the test. To measure passive ROM, the rater moved the joint to its end-range position without causing pain. To measure active ROM, the participant moved the joint to its end-range position and the rater asked the participant: “Is this your maximum range of motion?”

### Statistical analysis

Clinimetric properties and criterion validity (concurrent validity) were used to evaluate the validity of the self-reported 5PQ versus the BeS [33, 34]. The BeS is the most commonly used and recognised test for assessing GJH and was therefore used as the reference test [2, 4]. The validity of the self-reported 5PQ was tested versus a cut-off level of ≥5 for the BeS.

Data from the clinical measurements were manually entered into a web-based tool by the raters. The sociodemographic data and the self-reported 5PQ were entered into the web-based tool by the participants. Descriptive statistics were used for sociodemographic data. The normality of the distribution was investigated using graphs.

Spearman’s rho with 95% confidence intervals (CI) was used for evaluation of the correlation between the self-reported 5PQ and the BeS. The optimal combinations of cut-off levels for the population studied, giving the highest combined sensitivity and specificity with 95% confidence intervals (CI) of the self-reported 5PQ versus the BeS, were determined [35, 36].

A receiver operating characteristic (ROC) curve was created to illustrate the proportions of sensitivity and specificity for the test at different cut-off levels, in order to determine the most appropriate cut-off level for the self-reported 5PQ for the studied population. The ROC is based on cross-tabulation of categorical outcomes, where all possible cut-offs were studied. The most appropriate cut-off level was selected from the sensitivity since the 5PQ is used to assess people with GJH and is also used in research as exposure variable.

The area under the ROC curve (AUC) with 95% confidence intervals (CI) was calculated. The highest possible value of AUC is 1.0, and the higher the value, the better a test is at discriminating between positive or negative cases [36, 37].

To define the proportion of participants with a positive self-reported 5PQ who were correctly identified with GJH, a positive predictive value (PPV) was used. To define the proportion of participants with a negative self-reported 5PQ who were correctly identified without GJH, a negative predictive value (NPV) was used. PPV and NPV were calculated with 95% confidence intervals (CI). A value of 100% is considered to be perfect. The PPV and NPV depend on the GJH prevalence and were therefore adjusted with an estimated GJH prevalence of 10% estimated from a previous study within the general population in Sweden [19, 36, 38].

The positive likelihood ratio (LR+) was used, calculated with 95% confidence intervals (CI), to determine how many times more likely a person with GJH was to have a positive self-reported 5PQ than a person without GJH. LR+ above 10 is considered strong evidence predicting the presence or absence of a diagnosis [36, 39].

Fagan’s nomogram was used to illustrate how much a result from a diagnostic test changed the probability that a person had the diagnosis [40]. The post-test probability was calculated using an estimated GJH prevalence of 10% within the general population in Sweden as pre-test probability.

All joint angles included in the BeS were measured in degrees, with means calculated, and related to the different cut-off levels for the self-reported 5PQ and the BeS.

All analyses were carried out using Stata/IC 15.1 (StataCorp LLC, Texas, USA) and R version 3.5.2.

### Ethics

The study was approved by the Regional Ethical Review Board, Box 2110, 750 02 Uppsala, Sweden, dnr 2013/186. Department of Public Health and Caring Sciences, Uppsala University, Uppsala, Sweden.

## Results

Table 1 shows sociodemographic data and the distribution of the BeS and 5PQ among included participants. A total of 339 women fulfilled the sociodemographic data, with a mean (SD) age of 31 (4.4) years. Of these, 86.1% were born in Sweden.

**Table 1:** Sociodemographic characteristic of 339 women in early pregnancy

Variable		Missing
Age, mean in years (SD)	31.0 (4.4)	
Completed weeks of gestation, mean^a^ (SD)	12.2 (2.2)	4
BMI, mean (SD)	24.7 (4.4)	6
Married/cohabiting (%)	94.4	4
Completed University education (%)	67.6	4
Place of birth (%)		
- Sweden	86.1	6
No previous pregnancies (%)	37.8	3
No previous deliveries (%)	51.6	3
Non-smokers (%)	97.9	3
Beighton score ≥ 5 (%)	15.9	6
Self-reported 5PQ ≥ 2 (%)	40.4	33
Clinical assessment after answering 5PQ (%)	64.3	2

*SD* Standard deviation

^a^Estimated after ultrasound

*BMI* Body mass index. The body-mass index is the weight in kilograms divided by the square of the height in meters

*5PQ* Five-part questionnaire. Each positive response adds 1 point, with a total score ranging from 0 to 5

1. Can you now (or could you ever) place your hands flat on the floor without bending your knees?

2.Can you now (or could you ever) bend your thumb to touch your forearm?

3. As a child, did you amuse your friends by contorting your body into strange shapes or could you do the splits?

4. As a child or teenager, did you dislocate your shoulder or kneecap on more than one occasion?

5. Do you consider yourself double-jointed?

*BeS* Beighton. A positive result in any assessment adds 1 point, with a total score ranging from 0 to 9

1. Passive dorsiflexion of the fifth metacarpophalangeal joint ≥90 degrees

2. Passive apposition of the thumb to the flexor aspects of the forearm

3. Passive hyperextensions of the elbow ≥10 degrees

4. Passive hyperextensions of the knee ≥10 degrees

5. Forward flexion of the trunk with knees straight, with the palms of the hands rested on the floor

### Response analysis

Of the 339 participants, 306 completed the self-reported 5PQ, and 64.3% of these answered the self-reported 5PQ before their clinical assessment. Three participants did not fill out the questionnaire. One participant did not answer question 1. Six participants answered, do not remember, on question 1, six participants answered, do not remember, on question 2, eight participants answered, do not remember, on question 3 and six participants answered, do not remember, on question 4. Three participants answered only three out of five questions. These participants where therefore excluded from the analysis.

Of the 339 participants, 333 were assessed using the BeS. Two participants were not assessed. In one participant, the left and right elbow extensions were not assessed, in another, the left thumb was not assessed and in two participants the right thumb were not assessed. A total of 301 participants were included in the validity analyses.

In the self-reported 5PQ the most prevalent positive responses was, “Can you now (or could you ever) place your hands flat on the floor without bending your knees?” (52.5%). The question with fewest positive responses was, “As a child or teenager, did you dislocate your shoulder or kneecap on more than one occasion?” (6.5%).

The frequency of each item in the BeS ranged from 11 to 32%, with the most common abilities being left thumb apposition (32%) and palms to floor (29%).

Table 2 shows the cumulative frequency of total scores for the self-reported 5PQ and the BeS. Among the 306 participants answering the 5PQ, 137 (40.4%) had scores of ≥2 and 76 (22.4%) had scores of ≥3. Of the 333 participants assessed by the BeS, 54 (15.9%) had scores of ≥5.

**Table 2:** Cumulative frequency for 5PQ, *N* = 306, and BeS, *N* = 333, in different cut-off levels

	Frequency n (%)
**5PQ**	
**≥ 0**	306 (100)
**≥ 1**	227 (67.0)
**≥ 2**	137 (40.4)
**≥ 3**	76 (22.4)
**≥ 4**	32 (9.4)
**≥ 5**	4 (1.2)
**BeS**	
≥ **0**	333 (100)
≥ **1**	282 (83.2)
≥ **2**	179 (52.8)
≥ **3**	138 (40.7)
≥ **4**	81 (23.9)
≥ **5**	54 (15.9)
≥ **6**	36 (10.6)
≥ **7**	21 (6.2)
≥ **8**	2 (0.6)
≥ **9**	1 (0.3)

*5PQ* Five-part-questionnaire, *BeS* Beighton score

There was a positive correlation (*r* = 0.62, *p* < 0.001, CI 0.53–0.68) between the self-reported 5PQ and the clinically assessed BeS.

**Fig. 1:**
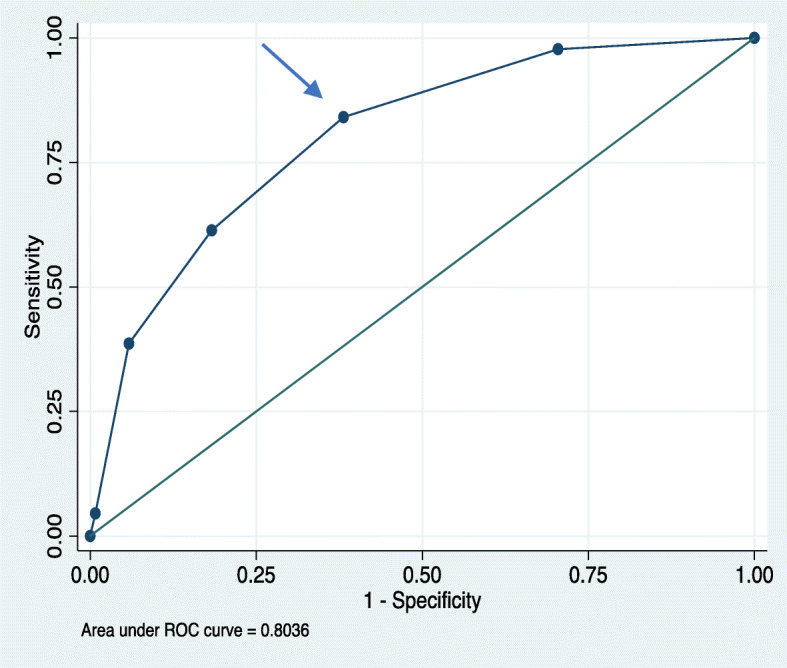
ROC; Receiver operating characteristic curve with true positive values (sensitivity) on the Y-axis and false positive values (1-specificity) on the X-axis. Cut-off level 1–5 on the self-reported five-part questionnaire, 5PQ, evaluated against the Beighton score ≥ 5. Arrow shoes cut-off level ≥ 2 on the 5PQ

After studying the ROC curve, the most appropriate cut-off level for the 5PQ was found to be ≥2, with an AUC of 0.73 (95% CI 0.67–0.79), Fig. 1.

**Fig. 2:**
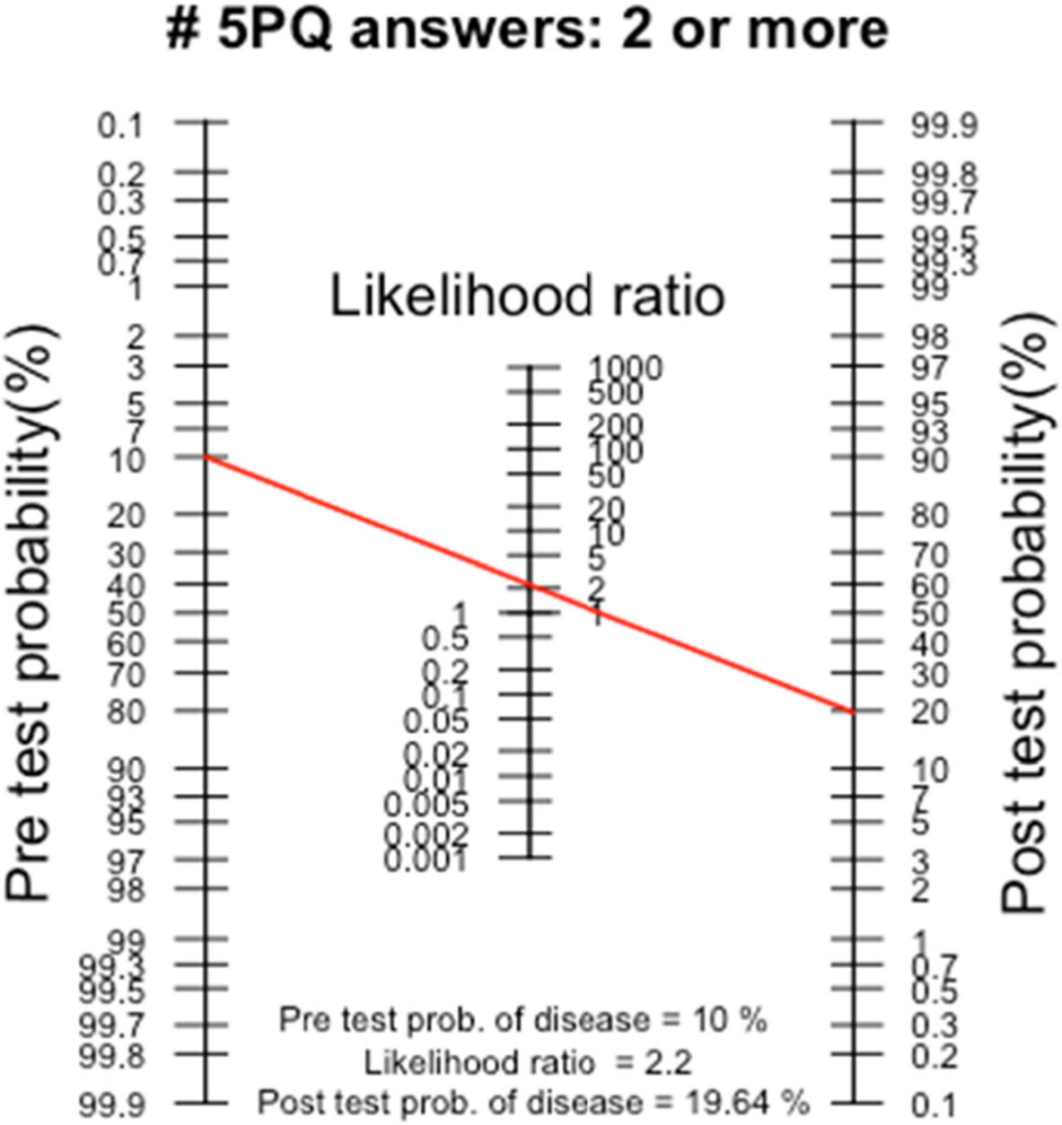
Fagan’s nomogram

Table 3 shows a cross tabulation result of index test, 5PQ ≥ 2, against reference standard, BeS ≥ 5. Table 4 shows the highest combined sensitivity, 84.1% (95% CI 69.9–93.4), and specificity, 61.9% (95% CI 55.6–67.8). The false-positive rate was 38%. The PPV and NPV ranged between 13.4 and 42.4% and between 90.3 and 99.2%, respectively. The LR+ increased by 2.2 when using a cut-off level of ≥2 for the self-reported 5PQ, which is illustrated with the Fagan’s nomogram in Fig. 2 showing a post-test probability of 19.6%.

**Table 3:** Cross tabulation of the 5PQ **≥ 2** and the BeS **≥ 5,** prevalence adjusted with 10%

	Positive BeS ≥ 5	Negative BeS < 5	Total
Positive 5PQ **≥ 2**	37	98	135
Negative 5PQ **< 2**	7	159	166
Total	44	257	301

*5PQ* Five-part-questionnaire, *BeS* Beighton score

**Table 4:** Diagnostic accuracy, 5PQ compared with the BeS cut-off ≥5. Prevalence adjusted with 10%

5PQ	Sensitivity % (CI)	Specificity % (CI)	AUC (CI)	PPV % (CI)	NPV % (CI)	LR+ (CI)
**≥ 1**	97.7 (88.0–99.9)	29.6 (24.1–35.6)	0.64 (0.60–0.67)	13.4 (12.3–14.4)	99.2 (94.4–99.9)	1.4 (1.3–1.5)
**≥ 2**	84.1 (69.9–93.4)	61.9 (55.6–67.8)	0.73 (0.67–0.79)	19.7 (16.7–23.1)	97.2 (94.6–98.6)	2.2 (1.8–2.7)
**≥ 3**	61.4 (45.5–75.6)	81.7 (76.4–86.2)	0.72 (0.64–0.79)	27.2 (20.8–34.6)	95.0 (92.9–96.5)	3.4 (2.4–4.8)
**≥ 4**	38.6 (24.4–54.5)	94.2 (90.6–96.7)	0.66 (0.59–0.74)	42.4 (28.4–57.7)	93.2 (91.6–94.6)	6.6 (3.4–12.3)
**≥ 5**	4.5 (0.6–15.5)	99.2 (97.2–99.9)	0.52 (0.49–0.55)	39.4 (8.6–81.8)	90.3 (89.8–90.9)	5.8 (0.8–40.4)

*5PQ* Five-part-questionnaire, *BeS* Beighton score, *CI* Confidence interval, *AUC* Area under curve, *PPV* Positive predictive value, *NPV* Negative predictive value, *LR+* Positive Likelihood ratio

Range of motion, measured in degrees, in joints included in the BeS are shown in Tables 5 and 6, presented as means and ranges. The higher the scores on the self-reported 5PQ and in the BeS, the higher the joint ROM except for the thumb apposition, where the joint ROM should decrease. The measured joint angles varied in each cut-off level.

**Table 5:** Joint mobility in degrees of the five-part questionnaire, cut-off 0–5, presented as mean and range

Joints	Five-part questionnaire cut-off levels					
	0 (***n*** = 79)	1 (***n*** = 90)	2 (***n*** = 61)	3 (***n*** = 44)	4 (***n*** = 28)	5 (***n*** = 4)
Fifth finger extension left	72 (47–98)	73 (20–97)	74 (35–99)	77 (45–95)	86 (70–115)	88 (78–92)
Fifth finger extension right	66 (39–106)	66 (20–95)	69 (30–102)	71 (45–95)	81 (60–97)	83 (69–92)
Thumb apposition left	36 (15–85)	28 (5–71)	26 (2–54)	22 (5–46)	13 (3–33)	14 (5–23)
Thumb apposition right	36 (12–80)	30 (5–66)	28 (3–53)	24 (5–55)	15 (0–35)	18 (15–22)
Elbow extension left	4 (0–12)	5 (0–20)	7 (0–17)	8 (0–20)	9 (1–27)	8 (0–14)
Elbow extension right	4 (0–13)	5 (0–15)	7 (0–18)	7 (0–17)	8 (1–15)	6 (2–9)
Knee extension left	3 (0–15)	4 (0–14)	4 (0–14)	6 (0–20)	8 (0–17)	10 (5–15)
Knee extension right	3 (0–19)	4 (0–13)	4 (0–15)	6 (0–25)	7 (0–14)	8 (3–14)

*n* Number of participants

**Table 6:** Joint mobility in degrees of the Beighton score, cut-off 0–9, presented as mean and range

Joints	The Beighton score cut-off levels									
	0 (***n*** = 51)	1 (***n*** = 103)	2 (***n*** = 41)	3 (***n*** = 57)	4 (***n*** = 27)	5 (***n*** = 18)	6 (***n*** = 15)	7 (***n*** = 19)	8 (***n*** = 1)	9 (***n*** = 1)
Fifth finger extension left	69 (47–85)	68 (30–91)	76 (37–95)	75 (40–99)	82 (50–93)	86 (57–103)	88 (56–98)	91 (72–115)	90 (90)	97 (97)
Fifth finger extension right	64 (40–85)	62 (27–87)	71 (35–95)	71 (42–95)	77 (40–92)	81 (48–112)	85 (64–106)	85 (67–95)	80 (80)	97 (97)
Thumb apposition left	36 (15–85)	36 (13–71)	28 (2–57)	21 (5–50)	14 (3–45)	14 (5–30)	12 (3–22)	13 (5–28)	10 (10)	9 (9)
Thumb apposition right	38 (14–80)	37 (15–67)	30 (5–50)	22 (5–50)	15 (0–39)	15 (0–30)	17 (3–45)	12 (5–21)	10 (10)	6 (6)
Elbow extension left	3 (0–8)	4 (0–12)	6 (0–16)	7 (0–20)	8 (0–20)	9 (0–15)	12 (4–19)	13 (5–27)	13 (13)	11 (11)
Elbow extension right	3 (0–9)	3 (0–10)	6 (0–15)	7 (0–16)	7 (0–16)	8 (0–15)	11 (3–17)	12 (5–18)	14 (14)	12 (12)
Knee extension left	2 (0–8)	3 (0–10)	4 (0–11)	5 (0–20)	5 (0–13)	8 (0–18)	9 (0–15)	10 (0–17)	13 (13)	12 (12)
Knee extension right	2 (0–7)	3 (0–10)	4 (0–11)	5 (0–25)	5 (0–14)	7 (0–15)	8 (0–19)	9 (0–14)	14 (14)	10 (10)

*n* Number of participants

## Discussion

There was a moderate correlation between the self-reported 5PQ and the BeS. The highest values of sensitivity, specificity and an acceptable area under curve were observed on cut-off level ≥ 2 in the 5PQ. The false-positive rate was 38% with low positive predictive value and high negative predictive value. The odds and the post-test probability having generalized joint hypermobility with a positive self-reported five-part questionnaire, using a cut-off level of ≥2, was low.

The mean for all joint angles measured in degrees increased with increased cut-off levels, both in the BeS and in the self-reported 5PQ with a significant variation for each cut-off level.

GJH is a complex clinical condition, for which identification requires a broad clinical assessment. Which joints to include, specific cut-off levels for each included joint, how the measurement of ROM in joints should be performed, and what cut-off levels to use for GJH are all points of an ongoing discussion [15, 16]. In diagnostic accuracy studies, the presence or absence of the condition is ideally determined using a “gold standard” assessment method [41]. There is no gold standard for assessing GJH; therefore, the use of criterion validity and the BeS as a reference test could be discussed. However, the BeS is the instrument most used and since 2017 there has been consensus in assessing GJH in hereditary connective tissue disorders with the BeS. To assess GJH in clinical and research settings, the BeS was therefore considered to be a reasonable “gold standard” [2].

Depending on which statistical method was used in the present study, the usability of the self-reported 5PQ differed. Using sensitivity and specificity, the ideal situation is that both are high, to maximise the discriminative ability. However, there is a certain trade-off between them. This is related to the severity of the diagnosis, whether the test should detect or exclude a disease, and the costs and suffering for the patient if misdiagnosed [42]. A cut-off level of ≥2 for the self-reported 5PQ had the highest combined sensitivity, specificity, and AUC when compared with the BeS cut-off level of ≥5. This is, in agreement with previous studies [29, 30] using a cut-off level of ≥4 and ≥ 5 respectively for the BeS. Although a cut-off level of ≥2 for the self-reported 5PQ demonstrated the highest combined sensitivity and specificity in this study, an associated risk of including a high rate of false positives cannot be eliminated. Furthermore, the sensitivity and specificity say nothing about the probability of presence or absence of GJH, only the test result is known [35, 36]. In clinical practice, it is important to know if a test result predicts the risk of having a diagnosis. Therefore, the assessment of predictive value and LR+ was used [36, 38]. The LR+, illustrated with the Fagan’s nomogram estimates, is rarely used in studies evaluating diagnostic tests [43].

The prevalence affects the PPV and NPV and our results are only useful for studies with the same population and prevalence [38]. There are no new, large population studies in Sweden, so the estimated GJH prevalence of 10% that was used here was based on a study from 1993 [19]. The prevalence of GJH depends on gender, age, ethnicity, how the test is performed, as well as the cut-off values used, which will differ between studies. The PPV decreases and the NPV increases if the prevalence is low [36, 38]. However, when calculating the PPV and the NPV, different prevalence values were tested (15 and 20%) and neither the PPV nor the NPV changed significantly.

Ideally, a high sensitivity is required for a test assessing people with GJH, or if used as an exposure variable in research, however, including a low false-positive rate and a high positive predictive value.

Possible advantages of the self-reported 5PQ are that it increases the ability to identify individuals with joint hypermobility, it is easy to use, it does not rely entirely on specific joints, and the questions take into account a possible history of joint hypermobility [27]. However, it is important to distinguish between identifying joint hypermobility using a questionnaire and assessing it clinically with regard to the degree of joint mobility and pathology in multiple joints. A more thorough assessment of joint hypermobility is suggested to identify which type of JH, e.g. GJH and to set up a rehabilitation and treatment plan [4, 12]. The assessment of the presence and degree of joint hypermobility should not be arbitrary.

In this study, the highest positive response rate to a single question in the self-reported 5PQ was to question 1, “Can you now (or could you ever) place your hands flat on the floor without bending your knees?”, which was in accordance with the study by Moraes et al. [29]. In the current study, the difference was 20% when the answers to question 1 were compared in the self-reported 5PQ with the clinical assessment of forward flexion of the trunk with knees straight and the palms of the hands rested on the floor. In this study the prevalence of GJH in the self-reported 5PQ was 40.4% with a cut-off level of ≥2, and 22.4% with a cut-off level of ≥3. Compared with the BeS cut-off level of ≥5, the prevalence of GJH in this study was 15.9%. In a recent study [30] using a cut-off level ≥ 2 on the self-reported 5PQ and ≥ 5 for the BeS in females with the same age as in the present study the prevalence of GJH was 38.2 and 11.8% respectively, which is in accordance with our study. Compared with the study by Moraes et al. [29] having included mixed genders and ages, the prevalence rates based on the self-reported 5PQ, with cut-off levels of ≥2 and 3, were 37 and 9.9% respectively [29]. In the study by McKay et al. [18] age, sex, and waist circumference in adults, were found to be the strongest predictors for joint flexibility. We are of the opinion that by including only women in fertile age, mainly born in Sweden and having normal weight, might not have affected the difference in prevalence found in this study. Differences between answering the 5PQ as opposed to a clinical joint assessment could be that some people had been able to do the test historically, while responding to a questionnaire often results in an overestimation of an individual’s ability. Alternatively, participants might also not have known how to perform the test properly. Finally, the ROM assessed in a questionnaire is subjectively estimated and is not always passively assessed when self-performed. Passive performance of ROM is needed to achieve an end feel, and the adjacent joint should be stabilised. All these aforementioned factors could have affected the prevalence of GJH. Maybe the cut-off value of ≥2 for the self-reported 5PQ, may have been to low for this group and should have been adjusted to at least ≥3. In addition, we did not differentiate between asymptomatic and symptomatic GJH in this study which also could have influenced the prevalence.

As this study is part of a longitudinal study of women during and 9 months after pregnancy, with the overall aim of investigating joint mobility and its relation to pain during pregnancy, the result is representative only for young women. The ROM in joints was measured in early pregnancy. Previous studies, using various methodologies, show no clear evidence that joint mobility increases in early pregnancy [44, 45].

Our ambition was that the clinical assessments would take place after the participants had answered the self-reported 5PQ. This was the case for 64.3% of participants. However, the raters were blinded to the answers when performing the clinical assessments.

The mean angle for all specific joints increased with increased cut-off levels, both in the BeS and in the self-reported 5PQ, which is in accordance with another study [24]. However, the joint angles measured in degrees varied significantly in the different cut-off levels. Because of this variation, there is possibly a need to further study age-and sex specific cut-offs for the specific joint angles measured in degrees that differentiate those with and without GJH. Perhaps those classified as having GJH should be at the extreme, the uppermost 5%, of joint mobility for their population as suggested in the study by Singh et al. [17]. This study confirmed the current cut-off level of ≥5 in the BeS, for young women, although a different joint measurement methodology was used. A further conclusion, in the study by Singh et al. was that when using the BeS when assessing GJH, age- and sex-specific BeS cut-off levels based on the uppermost 5% should be used.

There are several limitations in this study. The first is the use of the BeS as the reference test. However, this study is done pending further research on the assessment of GJH. Another limitation in the study could be the estimated prevalence of GJH in the population studied, as mentioned above. Diagnostic accuracy should be tested on a population including those with both mild and severe abnormality in regard to what the instrument is supposed to measure, in this case GJH [36]. In the studied population, 10.6% had scores of ≥4 for the self-reported 5PQ and 7.1% had scores of ≥7 for the BeS. This might be too low a prevalence of severe GJH, which may have affected the result.

Another limitation of this study was that the Swedish translation of the self-reported 5PQ was not scientifically validated as were performed in the studies by Moraes et al. and Glans et al. [29, 30]. In this study the 5PQ was translated by a native English-speaking physician, who is also fluent in the Swedish language and familiar with the terminology. The translation was discussed until a consensual version was achieved together with the co-workers of the project. However, after the clinical assessments were completed, it emerged that there was some uncertainty in relation to question 2: “Can you now (or could you ever) bend your thumb to touch your forearm?” Some participants answered the question negatively, even though they had a positive clinical test, implicating a lack of understanding on how to perform the test. This is in line with the results in the studies by Moraes et al. and Glans et al. [29, 30]. To promote understanding, the question should be rewritten or illustrated, as in the study by Moraes et al. [29]. Additionally, in this study, as well as in the studies by Moraes et al. and Glans et al. [29, 30] there was also difficulty when translating the word “double-jointed” in the last question in the questionnaire. In this study, the Swedish word for “agile” was used while Moraes et al. [29], used the Brazilian Portuguese word for “flexible” and Glans et al. [30] used the Swedish word for “to have joint hypermobility” in their studies. Overall, our translation did not differ significantly compared with the version of Glans et al., and we believe that it did not affect the prevalence of GJH.

Both previous studies did a test-retest as a first phase in their studies, showing moderate to almost perfect reliability for each item on the 5PQ [29, 30]. This study did not include a test-retest investigation of the 5PQ, which is a limitation since high validity requires high reliability.

The strengths of the study include the use of PPV, NPV, and LR+ to describe the clinical usability of the test. Also, including only young women could be a strength in studies of GJH, as GJH is more prevalent in this group. Another strength of this study was that the measurement of ROM in joints was performed in accordance with a structured protocol and by experienced raters. The same protocol was used in a previous reliability study of the BeS, the Hospital del Mar criteria, and the Contompasis score, which showed good inter- and intra-rater reliability [32]. The validity of a clinical measurement instrument is dependent not only on its validity, but also on the data quality [34]. This depends for example on the experience of the rater and the amount of attention paid by the participant in answering the questionnaire [34]. Although, since 2017 there has been a consensus about assessing GJH in hereditary connective tissue disorders with the BeS [2, 26], it has been performed in many different ways in different studies. How joint ROM is measured is not always clearly described, which hampers comparison of results with other studies and with clinical assessments. However, there is still a need for further discussion about the use of measuring instruments such as goniometers and whether adjacent joints should be stabilized.

GJH should always be considered in the differential diagnosis for patients with widespread pain and soft tissue lesions. It should be easy to screen for joint hypermobility, but the assessment of the degree of joint hypermobility is probably more complex. Until now, clinimetric research has been about assessing joint ROM and angular mobility. The current assessment methods might not be sufficient for determining the severity of joint hypermobility. Further research should also include the assessment of joint instability and joint translation, especially joint gliding in the transverse-or sagital plane, both in the spine and the extremity joints. There is maybe also a need to evaluate if other joints than those included in the BeS should be included in the assessment of GJH. However, a single error-free gold standard is probably not the way to establish presence or absence of GJH.

## Conclusions

There is uncertainty in identifying generalized joint hypermobility in young women using the self-reported five-part questionnaire with cut-off level ≥ 2 using the Beighton score cut-off level ≥ 5 as the reference test. The strength of the self-reported five-part questionnaire is that it rules-out women without generalized joint hypermobility.

The mean for all joint angles measured in degrees increased with increased cut-off levels, both in the BeS and in the self-reported 5PQ. There was, however, a significant variation for each cut-off level.

## Data Availability

The datasets used and/or analysed in the current study are available from the corresponding author on reasonable request.

## Abbreviations

GJH: Generalised joint hypermobility
ROM: Range of motion
BeS: Beighton score
5PQ: Five-part questionnaire
BMI: Body mass index
CI: Confidence interval
ROC: Receiver operating characteristic
AUC: Area under curve
PPV: Positive predictive value
NPV: Negative predictive value
LR+: Positive likelihood ratio
SD: Standard deviation

## References

[1] Grahame R (2008). Hypermobility: an important but often neglected area within rheumatology. Nat Clin Pract Rheumatol.

[2] Malfait F, Francomano C, Byers P, Belmont J, Berglund B, Black J (2017). The 2017 international classification of the Ehlers-Danlos syndromes. Am J Med Genet C Semin Med Genet.

[3] Gazit Y, Jacob G, Grahame R (2016). Ehlers-Danlos syndrome-hypermobility type: a much neglected multisystemic disorder. Rambam Maimonides Med J.

[4] Castori M, Tinkle B, Levy H, Grahame R, Malfait F, Hakim A (2017). A framework for the classification of joint hypermobility and related conditions. Am J Med Genet C Semin Med Genet.

[5] Castori M, Hakim A (2017). Contemporary approach to joint hypermobility and related disorders. Curr Opin Pediatr.

[6] Bulbena A, Gago J, Pailhez G, Sperry L, Fullana MA, Vilarroya O (2011). Joint hypermobility syndrome is a risk factor trait for anxiety disorders: a 15-year follow-up cohort study. Gen Hosp Psychiatry.

[7] Baeza-Velasco C, Pailhez G, Bulbena A, Baghdadli A (2015). Joint hypermobility and the heritable disorders of connective tissue: clinical and empirical evidence of links with psychiatry. Gen Hosp Psychiatry.

[8] Hakim AJ, Grahame R, Norris P, Hopper C (2005). Local anaesthetic failure in joint hypermobility syndrome. J R Soc Med.

[9] Scheper MC, Juul-Kristensen B, Rombaut L, Rameckers EA, Verbunt J, Engelbert RH (2016). Disability in adolescents and adults diagnosed with hypermobility-related disorders: a meta-analysis. Arch Phys Med Rehabil.

[10] Mogren IM (2006). BMI, pain and hyper-mobility are determinants of long-term outcome for women with low back pain and pelvic pain during pregnancy. Eur Spine J.

[11] Keer R, Simmonds J (2011). Joint protection and physical rehabilitation of the adult with hypermobility syndrome. Curr Opin Rheumatol.

[12] Engelbert RH, Juul-Kristensen B, Pacey V, de Wandele I, Smeenk S, Woinarosky N (2017). The evidence-based rationale for physical therapy treatment of children, adolescents, and adults diagnosed with joint hypermobility syndrome/hypermobile Ehlers Danlos syndrome. Am J Med Genet C Semin Med Genet.

[13] Simmonds JV, Herbland A, Hakim A, Ninis N, Lever W, Aziz Q, et al. Exercise beliefs and behaviours of individuals with Joint Hypermobility syndrome/Ehlers-Danlos syndrome - hypermobility type. Disabil Rehabil. 2019;41(4):445–55. 10.1080/09638288.2017.1398278. 10.1080/09638288.2017.1398278 29125009

[14] Remvig L, Engelbert RH, Berglund B, Bulbena A, Byers PH, Grahame R (2011). Need for a consensus on the methods by which to measure joint mobility and the definition of norms for hypermobility that reflect age, gender and ethnic-dependent variation: is revision of criteria for joint hypermobility syndrome and Ehlers-Danlos syndrome hypermobility type indicated?. Rheumatology.

[15] Remvig L, Flycht L, Christensen KB, Juul-Kristensen B (2014). Lack of consensus on tests and criteria for generalized joint hypermobility, Ehlers-Danlos syndrome: hypermobile type and joint hypermobility syndrome. Am J Med Genet A.

[16] Juul-Kristensen B, Schmedling K, Rombaut L, Lund H, Engelbert RH (2017). Measurement properties of clinical assessment methods for classifying generalized joint hypermobility-a systematic review. Am J Med Genet C Semin Med Genet.

[17] Singh H, McKay M, Baldwin J, Nicholson L, Chan C, Burns J (2017). Beighton scores and cut-offs across the lifespan: cross-sectional study of an Australian population. Rheumatology.

[18] McKay MJ, Baldwin JN, Ferreira P, Simic M, Vanicek N, Burns J (2017). Normative reference values for strength and flexibility of 1,000 children and adults. Neurology.

[19] Larsson LG, Baum J, Mudholkar GS, Srivastava DK (1993). Hypermobility: prevalence and features in a Swedish population. Br J Rheumatol.

[20] Beighton P, Solomon L, Soskolne CL (1973). Articular mobility in an African population. Ann Rheum Dis.

[21] Bulbena A, Duro JC, Porta M, Faus S, Vallescar R, Martin-Santos R (1992). Clinical assessment of hypermobility of joints: assembling criteria. J Rheumatol.

[22] Ferrari J, Parslow C, Lim E, Hayward A (2005). Joint hypermobility: the use of a new assessment tool to measure lower limb hypermobility. Clin Exp Rheumatol.

[23] Erkula G, Kiter AE, Kilic BA, Er E, Demirkan F, Sponseller PD (2005). The relation of joint laxity and trunk rotation. J Pediatr Orthop B.

[24] Smits-Engelsman B, Klerks M, Kirby A (2011). Beighton score: a valid measure for generalized hypermobility in children. J Pediatr.

[25] Junge T, Jespersen E, Wedderkopp N, Juul-Kristensen B (2013). Inter-tester reproducibility and inter-method agreement of two variations of the Beighton test for determining Generalised Joint Hypermobility in primary school children. BMC Pediatr.

[26] Juul-Kristensen B, Rogind H, Jensen DV, Remvig L (2007). Inter-examiner reproducibility of tests and criteria for generalized joint hypermobility and benign joint hypermobility syndrome. Rheumatology.

[27] Hakim AJ, Grahame R (2003). A simple questionnaire to detect hypermobility: an adjunct to the assessment of patients with diffuse musculoskeletal pain. Int J Clin Pract.

[28] Grahame R, Bird HA, Child A (2000). The revised (Brighton 1998) criteria for the diagnosis of benign joint hypermobility syndrome (BJHS). J Rheumatol.

[29] Moraes DA, Baptista CA, Crippa JA, Louzada-Junior P (2011). Translation into Brazilian Portuguese and validation of the five-part questionnaire for identifying hypermobility. Rev Bras Reumatol.

[30] Glans M, Humble MB, Elwin M, Bejerot S (2020). Self-rated joint hypermobility: the five-part questionnaire evaluated in a Swedish non-clinical adult population. BMC Musculoskelet Disord.

[31] Mokkink LB, Terwee CB, Patrick DL, Alonso J, Stratford PW, Knol DL (2010). The COSMIN study reached international consensus on taxonomy, terminology, and definitions of measurement properties for health-related patient-reported outcomes. J Clin Epidemiol.

[32] Schlager A, Ahlqvist K, Rasmussen-Barr E, Bjelland EK, Pingel R, Olsson C (2018). Inter- and intra-rater reliability for measurement of range of motion in joints included in three hypermobility assessment methods. BMC Musculoskelet Disord.

[33] Mokkink LB, Terwee CB, Patrick DL, Alonso J, Stratford PW, Knol DL (2010). The COSMIN checklist for assessing the methodological quality of studies on measurement properties of health status measurement instruments: an international Delphi study. Qual Life Res.

[34] de Vet HC, Terwee CB, Bouter LM (2003). Current challenges in clinimetrics. J Clin Epidemiol.

[35] Altman DG, Bland JM (1994). Diagnostic tests. 1: Sensitivity and specificity. BMJ.

[36] Eusebi P (2013). Diagnostic accuracy measures. Cerebrovasc Dis.

[37] Altman DG, Bland JM (1994). Diagnostic tests 3: receiver operating characteristic plots. BMJ.

[38] Altman DG, Bland JM (1994). Diagnostic tests 2: predictive values. BMJ.

[39] Deeks JJ, Altman DG (2004). Diagnostic tests 4: likelihood ratios. BMJ.

[40] Fagan TJ (1975). Letter: Nomogram for Bayes theorem. N Engl J Med.

[41] Terwee CB, Bot SD, de Boer MR, van der Windt DA, Knol DL, Dekker J, et al. Quality criteria were proposed for measurement properties of health status questionnaires. J Clin Epidemiol. 2007;60(1):34–42. 10.1016/j.jclinepi.2006.03.012 17161752

[42] Björk J. Praktisk statistik för medicin och hälsa. 1st ed. Liber AB; 2011. p. 249-265.

[43] Hayden SR, Brown MD (1999). Likelihood ratio: a powerful tool for incorporating the results of a diagnostic test into clinical decisionmaking. Ann Emerg Med.

[44] Cherni Y, Desseauve D, Decatoire A, Veit-Rubinc N, Begon M, Pierre F (2019). Evaluation of ligament laxity during pregnancy. J Gynecol Obstet Hum Reprod.

[45] Lindgren A, Kristiansson P (2014). Finger joint laxity, number of previous pregnancies and pregnancy induced back pain in a cohort study. BMC Pregnancy Childbirth.

